# Application of a novel Musculoskeletal Ultrasound Sum Score (MUSS) in the follow-up of patients with juvenile idiopathic arthritis

**DOI:** 10.1093/rheumatology/keag137

**Published:** 2026-03-23

**Authors:** Daniel Windschall, Hatice Adiguzel-Dundar, Faekah Gohar

**Affiliations:** Clinic of Paediatric and Adolescent Rheumatology, St.-Josef-Stift Sendenhorst, Northwest German Center for Rheumatology, Sendenhorst, Germany; Medical Faculty Martin-Luther-University Halle-Wittenberg, Halle, Germany; Clinic of Paediatric and Adolescent Rheumatology, St.-Josef-Stift Sendenhorst, Northwest German Center for Rheumatology, Sendenhorst, Germany; Clinic of Pediatric Rheumatology, University of Health Sciences, Behcet Uz Pediatric Diseases and Surgery Training and Research Hospital, Izmir, Turkey; Clinic of Paediatric and Adolescent Rheumatology, St.-Josef-Stift Sendenhorst, Northwest German Center for Rheumatology, Sendenhorst, Germany

**Keywords:** juvenile idiopathic arthritis, ultrasound score, disease activity

## Abstract

**Objective:**

The objective of this study was to evaluate the novel Musculoskeletal Ultrasound Sum Score (MUSS) as a longitudinal marker of disease activity (DA) in patients with JIA within a treat-to-target management approach.

**Methods:**

Demographics, clinical, laboratory and US (B Mode, BM; Power Doppler Ultrasound, PDU; scored 0–3, using the paediatric OMERACT score) findings were recorded over a 1-year follow-up in the PRO-KIND cohort. The US imaging of joints was individual to the patient, determined by the treating clinicians (unaware of the study aims) as routine. MUSS (a combination of the highest single BM grade of all examined joints + the highest single PDU grade of all examined joints) was calculated (maximum score 6). Spearman correlations with clinical activity measures and ROC-derived MUSS cut-offs for clinical inactive disease were calculated, using pooled visit-level data.

**Results:**

Thirty-three JIA patients were included (oligoarthritis: *n* = 12, median age 4.4 years; polyarthritis: *n* = 21, 5.3 years). The median baseline MUSS was similar between subtypes. MUSS values decreased significantly over follow-up, paralleling improvements in JADAS-10 and global assessment scores. MUSS correlated strongly with JADAS-10, physician and parent/patient global scores (*Ρ* = 0.68–0.81; all *P* < 0.001) and with ESR in polyarthritis (*Ρ* = 0.57; *P* < 0.001). ROC analysis identified a MUSS cut-off of ≥1 as optimal for detecting active disease (specificity 100%, sensitivity 55%).

**Conclusion:**

MUSS is a promising, feasible sonographic score for longitudinal monitoring of disease activity in JIA, showing strong correlations with clinical measures and excellent specificity for active disease at a cut-off of ≥1. Validation in independent, prospective cohorts is warranted.

Rheumatology key messages

- The Musculoskeletal Ultrasound Sum Score (MUSS) was evaluated as a longitudinal ultrasound disease activity marker.
- MUSS is calculated from the sum of the highest single B Mode and Power Doppler grade.
- MUSS improved in parallel with clinical disease activity over follow-up.

## Introduction

JIA is the most common chronic rheumatic disease in children, characterized by joint inflammation that can lead to pain, swelling and irreversible bone damage. Timely and personalized monitoring of disease activity is essential for optimizing management and preventing long-term sequelae in affected children [1].

Musculoskeletal US (MSUS) is a valuable, non-invasive imaging tool for assessing joint inflammation in JIA. It enables visualization of soft tissues, synovial effusion, synovial hypertrophy, intrasynovial hypervascularization and early erosive changes that may not be evident on clinical examination or conventional radiography. Subclinical synovitis has been shown to occur frequently in asymptomatic JIA patients [2–4].

For the standardized documentation of disease activity, recent international initiatives have developed validated US scores, scoring systems, or examination protocols for specific joints [5–7]. The semi-quantitative OMERACT grading system for B-mode (BM) and Power Doppler Ultrasound (PDU) was established [8] and later adapted for paediatric populations [9]. While effective for single-joint evaluation, semi-quantitative scores may vary according to the imaging plane or between joints within the same patient. This was also demonstrated in a recently published study by Sande *et al*., in which increasing PDU grades were associated with higher BM grades and clinical arthritis [10]. Strategies that summarize inflammation across multiple joints can provide a broader picture yet may be time-consuming for routine use. Alternatively, the grey-scale plus Power Doppler (GSPD) ‘indicator joint’ method uses the most severely affected joint to represent disease activity but may not capture the total inflammatory burden [11].

The Musculoskeletal Ultrasound Sum Score (MUSS) was proposed to address these limitations by summing the highest single BM score and the highest single PDU score from all examined joints, potentially capturing maximal inflammatory activity while remaining feasible for clinical practice. Unlike GSPD, which is derived from both the BM and PDU grade from the same joint, MUSS allows these maximum scores to originate from different joints, accommodating joint-specific differences in US findings.

Despite the increasing integration of MSUS into JIA management, few studies have longitudinally assessed sonographic scores alongside clinical and laboratory markers of disease activity under treatment [12, 13]. Such studies are crucial within the treat-to-target framework, in which responsive imaging markers can guide individualized therapy. The PRO-KIND prospective, multicentre treat-to-target study enrolled patients with JIA from diagnosis and included standardized MSUS examinations at regular intervals [14].

The study therefore aimed to perform a longitudinal analysis of changes in the novel MUSS compared with other clinical and laboratory disease activity measures in patients with oligoarticular and polyarticular JIA under treatment. It was hypothesized that the MUSS, defined as the sum of the highest single BM and PDU scores across all scanned joints, would correlate with established clinical and laboratory markers and serve as a reliable longitudinal measure of sonographic disease activity.

## Methods

### Patient inclusion

Patients with a new diagnosis of JIA, oligoarthritis or seronegative polyarthritis (including patients with extended oligoarthritis) defined according to the ILAR classification criteria who consented to participation in the prospective PRO-KIND cohort were included for analysis. Data on demographic and disease characteristics collected from the included patients comprised age at diagnosis, gender, and diagnosis subtype. At each follow-up, laboratory values (CRP and ESR) and clinical disease activity parameters [active joint count, physician global assessment (PhGA), patient/parent global assessment (PGA), and JIA Disease Activity Score (JADAS-10) [14]] were recorded. The JADAS-10 is a composite score comprised of the sum of the active joint count (maximum 10), normalized ESR (ESR-20/10), PhGA and PGA, with scores ranging from 0 to 40 (higher scores indicating more severe disease activity). MSUS parameters, including BM and PDU scores using the paediatric OMERACT score, required for calculating the MUSS, were also documented.

The included patients participated in the PRO-KIND cohort from the time of diagnosis, which was defined as the baseline (Time 0, T0), for analysis. Follow-up visits were scheduled approximately every 3 months during the first year (T1, T2, T3). Ultrasound data were collected retrospectively from patient records and the image archiving system as part of routine standard clinical follow-ups.

### Musculoskeletal ultrasound protocol

MSUS scans were performed by experienced paediatric rheumatologists with at least 3 years of experience in MSUS. Measurement and documentation of BM and PDU was performed as standard procedure in the clinical notes, independent of the study aims. Clinicians were not aware of the aim of analysing the MUSS score, which was first calculated retrospectively by the study team at the conclusion of the study and checked by two independent members. Patients were scanned according to the local protocol, which at baseline usually includes examination of both knees, ankles (tibiotalar and subtalar), and midfoot [talo-navicular (TN) and calcaneo-navicular (CN)] regions and in polyarthritis additionally both hips, wrists, and elbows. Other joints are usually examined based on clinical indication, independent of the study. With the aim of evaluating a typically performed US protocol, the results from the acromioclavicular, sternoclavicular and temporomandibular joint scans were excluded from our MUSS calculation. All joints were scanned bilaterally as standard. All US evaluations were performed on a Canon Aplio i800 or Aplio A system using multifrequency linear transducers with a frequency range of 6–24 MHz (Canon Medical Systems, Tokyo, Japan). Doppler settings were 5.3–7 MHz Doppler frequency, 0.6 kHz pulse repetition frequency, and Colour Gain at 40–45 adapted to artifacts. In cases with BM positivity (minimum grade 1), PDU was assessed. US reporting included evaluation of synovial hypertrophy and effusion (BM) and intra-synovial blood flow (PDU), recorded qualitatively and semi-quantitatively. The previously published semi-quantitative paediatric OMERACT score, graded from 0 to 3 based on severity, was used to evaluate the MSUS findings [9]. MSUS results were recorded for each individual joint scanned per time point. The highest BM and highest PDU of any evaluated joint (not necessarily the same joint) were summed to form the MUSS for each patient examination. The highest possible MUSS was 6 (maximum 3 for BM and 3 for PDU), and the minimum score was 0.

### Statistical analysis

Analyses were performed using IBM SPSS version 25 (IBM Corp., Armonk, NY, USA). Demographic, disease and sonography data were analysed using descriptive statistics. Frequencies (percentages) were reported for categorical variables and medians (interquartile ranges) for continuous variables without normal distribution. Data distribution was assessed using the Shapiro–Wilk test. For categorical variables, the chi-squared test or Fisher’s exact test was used, as appropriate. Continuous variables were analysed using Student’s *t* test or the Mann–Whitney *U* test, depending on normality. For BM and PDU analysed as categorical variables, a cut-off of >1 was used to define positivity. A *P*-value of <0.05 was considered statistically significant.

At baseline (T0), BM, PDU and MUSS values showed approximately normal distribution and were therefore summarized as mean ± s.d., whereas at follow-up visits (T1–T3) these variables demonstrated a highly skewed distribution with a floor effect and were therefore presented as medians with corresponding ranges.

Correlations between MUSS and clinical disease activity measures were calculated using Spearman’s rank correlation coefficient. For this analysis, all available visits over the 1-year follow-up were pooled at the visit level, and separate analyses were performed for oligoarthritis (37 visits) and polyarthritis (81 visits). MUSS values were correlated with the JADAS-10 total score, PhGA, PGA and ESR. The strength of the correlation coefficients was interpreted as follows: *Ρ* < 0.3 weak, 0.3–0.5 moderate, 0.5–0.7 strong, and >0.7 very strong.

Receiver operating characteristic (ROC) curve analyses were performed using the pooled visit-level dataset (T0–T3), with separate models for oligoarthritis and polyarthritis. CID was defined using the Backström *et al.* JADAS-10 thresholds (<0.5 for oligoarthritis and <0.7 for polyarthritis) [15]. Optimal cut-off values were identified using the maximum Youden index. Area under the curve (AUC) values were interpreted as follows: 0.90–1.00 excellent, 0.80–0.90 good, 0.70–0.80 moderate, 0.60–0.70 poor, and 0.50–0.60 unsuccessful.

## Results

### Patient disease and demographic characteristics at baseline

Thirty-three patients with oligoarthritis (oligoarthritis, *n* = 12) or polyarthritis (*n* = 21; oligoarthritis-extended, *n* = 3; seronegative polyarthritis, *n* = 18) were included at baseline (T0). In the oligoarthritis group, 9/12 (75%) were female with a median age of 4.4 years (IQR 2.1). All were rheumatoid factor (RF) negative at inclusion; HLA-B27 was negative in 3/12 patients (not measured in 9/12). In the polyarthritis group, 16/21 (76%) were female and 2/8 were HLA-B27 positive (6/8 untested), with a median age at diagnosis of 5.3 years (IQR 6.7).

Baseline inflammatory markers were: CRP median 0.0 mg/dl (range 0–1.7) in oligoarthritis and 0.4 mg/dl (0–7.4) in polyarthritis; ESR median 16 mm/h (range 5–80) and 34 mm/h (5–75) in oligoarthritis and polyarthritis, respectively. Baseline JADAS-10 scores were median 11 (range 8–20) in oligoarthritis and 22.5 (10–31) in polyarthritis. PhGA and PGA were 4.9 ± 1.4 and 4.6 ± 1.4 in oligoarthritis, and 6.4 ± 1.6 and 6.3 ± 2.0 in polyarthritis, respectively.

### Follow-up visits and disease activity

Follow-up attendance and timing were as follows for oligoarthritis: T1 (median 3.0 months, IQR 0): 9/12 (75%); T2 (8.0 months, IQR 1.1): 7/12 (58%); T3 (12.0 months, IQR 1.0): 10/12 (83%). Though 9/12 attended at T1, only 8/12 patients had ultrasound examination performed as one patient attended with a non-related infection and as they reported no musculoskeletal symptoms, were discharged without ultrasound examination after the unremarkable clinical examination. For polyarthritis, findings for attendance were: T1 (median 4.0 months, IQR 1.0): 21/21 (100%); T2 (7.0 months, IQR 2.0): 18/21 (86%); T3 (12.0 months, IQR 3.0): 21/21 (100%).

**Figure 1:**
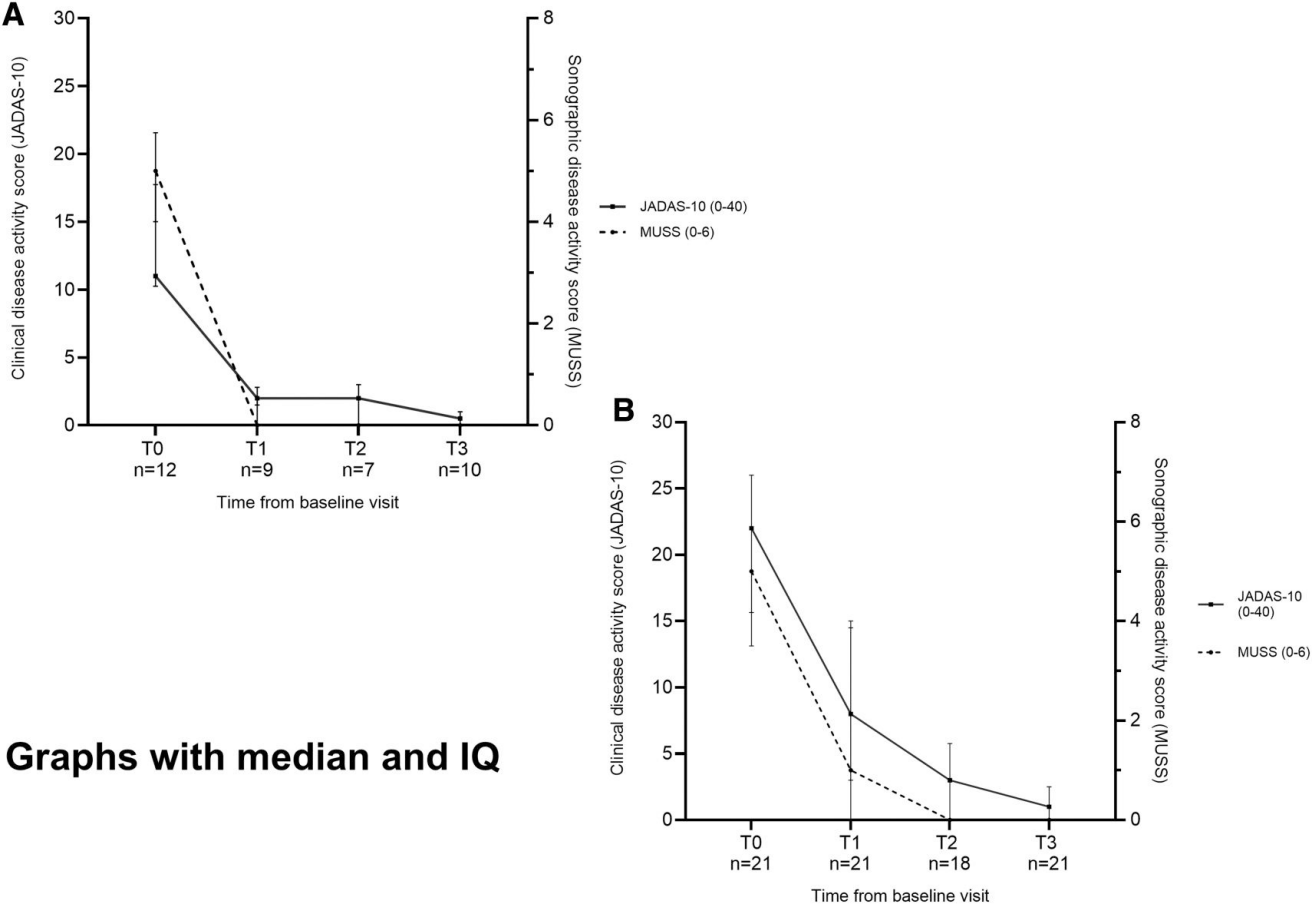
Change in sonographic (MUSS) and clinical disease activity (JADAS-10) scores from baseline to 1-year follow-up in patients with oligoarticular or polyarticular JIA. Graph shows change in the clinical disease activity score (JADAS-10) and sonographic score (MUSS) over time from baseline to 1 year after diagnosis of JIA in patients with (**A**) oligoarticular JIA or (**B**) polyarticular JIA. The JADAS-10 (score range 0–40) is indicated by the continuous line, and the MUSS score (range 0–6) is represented by the dotted line. The median and interquartile range for each score at each time-point are shown, and the ‘*n*’ patients included per time-point is indicated on the *x-*axis (oligoarticular JIA: maximum *n* = 12, polyarticular JIA: maximum *n* = 21). JADAS-10: Juvenile Arthritis Disease Activity Score-10; MUSS: Musculoskeletal Ultrasound Sum Score

In total, 37/48 (77%) possible visits were analyzed for oligoarthritis and 81/84 (96%) for polyarthritis. At each follow-up, median CRP and ESR values remained within the normal range in both groups. Clinical disease activity measures (PhGA, PGA, active joint count, JADAS-10) decreased after baseline in both cohorts, with the greatest improvement between T0 and T1 and further improvement or stability thereafter (Supplementary Table S1). MUSS and JADAS-10 changed in parallel over time (Fig. 1).

### Medication use

At baseline, 11/12 (92%) and 19/21 (91%) of patients were started on methotrexate therapy in the oligo- and polyarthritis groups respectively. The two patients without methotrexate were patients with oligoarthritis (later, oligoarthritis-extended). From T1 to T3 all patients in the oligoarthritis group were treated with methotrexate, whilst none received biological therapy at any time. In the polyarthritis group, treatment during follow-up was as follows: T1: 20/21 (95%) methotrexate and 8/21 (38%) TNF-inhibitor therapy, T2: 18/18 (100%) methotrexate and 11/18 (61%) TNF-inhibitor therapy, T3: 21/21 (100%) methotrexate and 11/21 (52%) TNF-inhibitor therapy. None of the patients were treated with steroid pulse therapy, however all patients received steroid joint injections for affected joints at baseline.

### Ultrasound investigation at baseline

**Figure 2:**
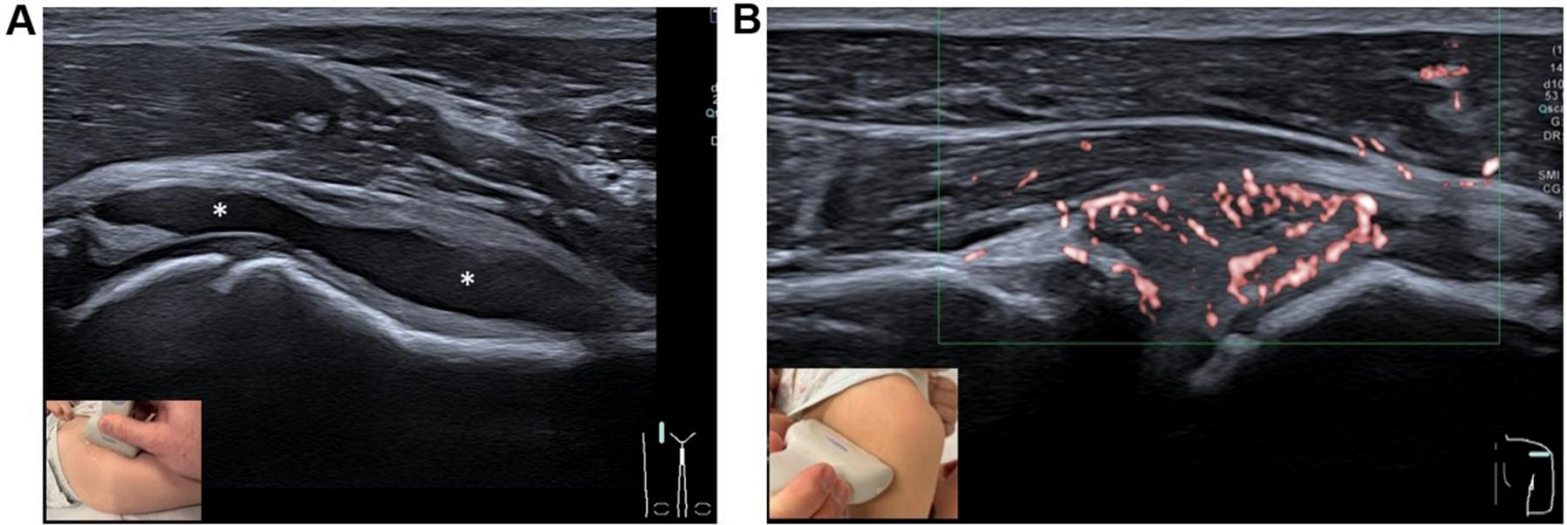
An example of B-Mode and power Doppler grade 3 in two joints with active arthritis. **(A)** An example of the sonography ventral longitudinal view of a hip joint with B-Mode Score Grade 3: a large significant effusion (asterisks) that leads to the development of a convex-shaped anterior capsula can be seen with extension of the effusion to the epiphysis. **(B)** An US image of the dorsal transverse view of the glenohumeral joint of the shoulder. Confluent intrasynovial power Doppler signals in over 30% of synovial tissue can be seen, representing an example of a joint with a power Doppler Score Grade 3 when graded using the paediatric OMERACT score

At T0, 112 joints (56 bilaterally) were scanned in oligoarthritis and 248 joints (124 bilaterally) in polyarthritis. Knees, ankles and midfoot regions were scanned in all patients. In polyarthritis, both hips were scanned in all patients, while 17/21 (81%) also had both elbows and wrists scanned. In oligoarthritis, hips, elbows and wrists were scanned in 8/12 (67%), 6/12 (50%) and 5/12 (42%) patients, respectively. Shoulders were infrequently scanned [oligoarthritis: 1/12 (8%); polyarthritis: 6/21 (29%)]. Baseline mean±SD BM, PDU and MUSS scores were: oligoarthritis: BM 2.5 ± 0.6, PDU 1.9 ± 1.0, MUSS 4.4 ± 1.5; polyarthritis: BM 2.4 ± 0.6, PDU 1.8 ± 1.0, MUSS 4.2 ± 1.2 (Fig. 2). The highest BM and highest PDU scores contributing to the MUSS calculation for each patient are listed in Supplementary Table S2 (oligoarthritis) and S3 (polyarthritis).

### Ultrasound during follow-up

Across all time points, 314 joints (mean 7 joints/patient/visit) were scanned in oligoarthritis and 818 joints (mean 10 joints/patient/visit) in polyarthritis. BM, PDU and MUSS decreased over time (Supplementary Table S4). By T1 in oligoarthritis, median BM, PDU and MUSS were already <0.2, reaching 0 by T2–T3. In polyarthritis, T1 values were reduced by at least 50% from baseline (BM 0.9 ± 1.1, PDU 0.8 ± 1.0, MUSS 1.8 ± 2.1), with minimal residual activity at T3 (BM 0.1 ± 0.5, PDU 0.0 ± 0.2, MUSS 0.2 ± 0.6).

### Correlation of MUSS with clinical parameters

MUSS correlated significantly with JADAS-10, PhGA, and PGA in both oligoarthritis (*Ρ* = 0.79, 0.80, 0.68; all *P *<  0.001) and polyarthritis (*Ρ* = 0.81, 0.80, 0.74; all *P *< 0.001). MUSS also correlated with ESR in polyarthritis (*Ρ* = 0.57, *P *< 0.001) but not in oligoarthritis (Table 1). These correlations were calculated using the pooled visit-level dataset (T0–T3).

**Table 1:** Correlation between MUSS and clinical disease activity measures in oligoarthritis and polyarthritis patients across follow-up visits.

	Oligoarthritis (37 visits)		Polyarthritis (81 visits)	
Correlation between MUSS	*Ρ*, rho	*P* value	*Ρ*, rho	*P* value
JADAS-10 score (range 0–40)	0.792	<0.001	0.813	<0.001
Physician global assessment (range 0–10)	0.796	<0.001	0.791	<0.001
Patient/Parent global assessment (range 0–10)	0.681	<0.001	0.737	<0.001
ESR (mm/h)	0.340	0.167	0.571	<0.001

Spearman’s rho correlation coefficient. *P *< 0.05 was considered statistically significant. JADAS-10: Juvenile Arthritis Disease Activity Score-10; MUSS: Musculoskeletal Ultrasound Sum Score; PGA: patient/parent global assessment; PhGA: physician global assessment.

### Diagnostic accuracy of MUSS

Clinical inactive disease (CID) was defined according to the criteria defined by Wallace *et al.* [16]. The more stringent JADAS-10 thresholds proposed by Backström *et al.* [17] (JADAS-10 < 0.5 for oligoarthritis; <0.7 for polyarthritis) were applied, which are also stricter than those defined by Consolaro *et al.* (<1.0 for both subtypes) [18]. ROC analysis showed good discrimination between CID and active disease with AUC 0.77 (95% CI: 0.61–0.93) for oligoarthritis and 0.78 (95% CI: 0.66–0.89) for polyarthritis (*P *< 0.001 for both). The optimal MUSS cut-off (≥1) yielded 100% specificity and 54% sensitivity for oligoarthritis and 100% specificity with 55% sensitivity for polyarthritis (Table 2). All estimates were derived from the pooled visit-level data.

**Table 2:** Diagnostic accuracy of Musculoskeletal Ultrasound Sum Score (MUSS) for the differentiation of sonographic active disease *vs* Clinical Inactive Disease (CID)

Region	AUC	AUC-ROC 95% CI	Cut-off MUSS	Sensitivity	Specificity	*P*	LR+	LR−	PV+	PV−	Youden index
**Oligoarthritis**											
	0.77	(0.61–0.93)	≥1	0.54	1.00	<0.001	∞	0.46	100	40.9	0.54
**Polyarthritis**											
	0.78	(0.66–0.89)	≥1	0.55	1.00	<0.001	∞	0.45	100	31.8	0.55

Cut-off value (≥1) derived from ROC analysis for discriminating CID from active disease according to JADAS-10 thresholds (<0.5 for oligoarthritis, <0.7 for polyarthritis). Less than the cut-off indicates no arthritis. Greater than or equal to the cut-off value indicates arthritis. AUC-ROC: area under the receiver operator characteristic curve; CID: clinical inactive disease. LR: likelihood ratio (cut-off: oligoarthritis < 0.5, polyarthritis < 0.7). MUSS: Musculoskeletal Ultrasound Sum Score; PV: predictive value.

## Discussion

In total, 33 patients with oligo- or polyarticular JIA were followed-up from baseline to 1 year. Routinely performed MSUS showed longitudinal improvement of the BM ultrasound, PDU and resulting MUSS parameters, which also correlated with clinical DASs. A MUSS cut-off of ≥1 was identified by ROC analysis as optimal for distinguishing active disease from CID, when CID was defined using stringent JADAS-10 cut-offs (<0.5 for oligoarthritis, <0.7 for polyarthritis). This threshold demonstrated perfect specificity but moderate sensitivity, suggesting that a MUSS of ≥1 strongly indicates active disease, whereas a MUSS of <1 is highly consistent with CID. To our knowledge, this is the first longitudinal study to evaluate a novel US activity score in both oligoarticular and polyarticular JIA under routine treatment, providing evidence that MUSS is a practical, responsive imaging marker for disease monitoring.

Patients with polyarthritis attended follow-ups more frequently (96% *vs* 77%) and had more joints scanned per follow-up, with more frequent scanning of the elbows, wrists and hips compared with patients with oligoarthritis, reflecting the usual joint involvement in these JIA subgroups. Patients with polyarthritis also had higher median ESR, affected joint count, PhGA, PGA, and JADAS-10 compared with oligoarthritis. The MUSS could be scored easily and practically, requiring only adequate reporting of BM and PDU grading, which is routine in our clinic. MUSS was not significantly different between the polyarticular and oligoarticular cohort at baseline, suggesting the MUSS could identify active sonographic disease independently of the specific JIA category. However, during follow-up, MUSS remained positive for longer in patients with polyarticular JIA compared with oligoarthritis, reflecting the longer persistence of disease activity as also measured by the JADAS-10 score in this group, who are more likely to require escalation to biologic therapy. In both groups, MUSS decreased significantly under treatment from baseline and correlated well with JADAS-10 and PGA, paralleling findings reported by Huang et al. [11] and Zhou et al. [19]. In contrast, Magni-Manzoni *et al.* found no correlation between the US severity score and clinical measures [20], whereas Collado *et al.* [13] proposed a reduced 10-joint US examination for sensitive monitoring of treatment response after 6 months. While oligoarthritis patients attended the T2 follow-up in low numbers, the changes in MUSS and JADAS-10 were already low and insignificantly different between T1 and T3, resulting in the assumption that results were indeed similar at T2, as patients with increased disease activity would be more likely to re-attend, though this cannot be ruled out. However, all inferential analyses (Spearman correlations and ROC models) were performed on pooled visit-level data (T0–T3), with each visit contributing an independent observation. Missing visits were not imputed, and because we did not conduct visit-specific inferential analyses, the smaller number of T2 attendees only affected the descriptive follow-up rates, not the correlation or ROC outcomes.

Interrater variability is a limitation of US evaluation, though previous studies from our group have shown good agreement [21]. Clinical disease activity scores such as JADAS-10, PGA and PhGA also contain subjective components, and physician–parent agreement on remission is imperfect [22–23]. The correlation of MUSS with these measures supports it as a sensitive imaging marker reflecting longitudinal change in disease activity, independently of the number of affected joints. However, we did not assess the sensitivity of MUSS for predicting flares, and previous studies suggest baseline US has low predictive value for future flares [24]. MRI confirmation was not performed due to its limited feasibility for multi-joint and repeated evaluations.

A mild BM grade 1 effusion can be a normal finding in healthy children [25], and US-detected synovial abnormalities are common in JIA remission [26–28]. In our cohort, the MUSS score normalized rapidly under treatment and showed minimal BM positivity after the first follow-up, closely aligning with the JADAS-10 score. Future research should evaluate whether a stricter cut-off (e.g. MUSS >1, reflecting BM grade 2 or BM grade 1 plus PDU grade 1) better defines active disease and avoids over-interpretation of small, non-pathological effusions. In clinical practice, a MUSS of ≥1 could serve as a supportive criterion for treatment escalation, complementing clinical and laboratory data within a treat-to-target framework.

This study has limitations, including its retrospective design and the lower number of oligoarticular JIA patients. Nevertheless, MUSS appears to be a promising, robust, and easy-to-implement sonographic score for monitoring disease activity, with performance characteristics that merit validation in larger, prospective and multicentre cohorts.

## Conclusion

The novel MUSS score showed strong correlations with established clinical measures of disease activity and demonstrated an optimal cut-off (≥1) for discriminating active disease from CID when defined by stringent JADAS-10 thresholds. Its ease of calculation and responsiveness to change make it a practical imaging tool for longitudinal monitoring of JIA within a treat-to-target approach. Although retrospective in nature, the findings support further validation in prospective, independent cohorts to confirm its diagnostic accuracy and clinical utility.

Parents and patients provided informed consent for participation in the PRO-KIND cohort study. Ethical approval was obtained from the local ethics commission, the Ärztekammer Westfalen-Lippe for the study titled ‘ProKind—Zusatzmodul zur Kerndokumentation rheumakranker Kinder und Jugendlicher’ in December 2020.

## Supplementary Material

keag137_Supplementary_Data

## Data Availability

The data underlying this article are available in the article and in its online supplementary material.
