# Supplementary material for: Application of a novel Musculoskeletal Ultrasound Sum Score (MUSS) in the follow-up of patients with juvenile idiopathic arthritis
Source: Rheumatology (Oxford). 2026 Mar 23;65(4):keag137. doi: 10.1093/rheumatology/keag137 (PMC13069893; doi:10.1093/rheumatology/keag137)
Supplement: keag137_Supplementary_Data [file keag137_supplementary_data.docx]

**Supplementary material**

**Supplementary Table S1**

Characteristics of the oligo- and polyarthritis patient group

|  | **T0 (Baseline)** | **T1** | **T2** | **T3** |
| --- | --- | --- | --- | --- |
| **Oligoarthritis** | | | | |
| CRP (mg/dL), n (%)  mean±SD  Median (min-max) | 12 (100)  0.4±0.6  0 (0-1.7) | 9 (75)  * normal (9/9)  0 (0) | 7 (58)  * normal (7/7)  0 (0) | 10 (83)  * normal (10/10)  0 (0) |
| ESR (mm/h), n (%)  mean±SD  Median (min-max) | 11 (92)  22.2±21.7  16(5-80) | 7 (58)  10.4±5.7  9(5-22) | 7 (58)  * normal (7/7)  (< 15) | 10 (83)  * normal (10/10)  (< 15) |
| Physician PGA (0-10), n (%)  mean±SD  Median (min-max) | 12 (100)  4.9±1.4  4.5(3-8) | 9 (75)  1.2±0.8  1(0-2) | 7 (58)  1.1±1.4  1(0-4) | 10 (83)  0.4±0.5  0(0-1) |
| Patient/Parent PGA (0-10), n (%)  mean±SD  Median (min-max) | 12 (100)  4.6±1.4  4(3-8) | 9 (75)  0.8±1.2  1(0-4) | 7 (58)  1.0±1.4  1(0-4) | 10 (83)  0.3±0.6  0(0-2) |
| JADAS-10, n (%)  mean±SD  Median (min-max) | 12 (100)  13.1±4.0  11(8-20) | 9 (75)  2.1±1.6  2(0-6) | 7 (58)  2.1±2.8  2(0-8) | 10 (83)  0.7±0.9  0(0-3) |
|  |  |  |  |  |
| **Polyarthritis** | | | | |
| CRP (mg/dL), n (%)  mean±SD  Median (min-max) | 21 (100)  1.6±2.1  0.4 (0-7.4) | 18 (86)  0.2±0.2  0.1 (0.0.8) | 18 (86)  * normal (18/18)  0 (0) | 18 (86)  * normal (18/18)  0 (0) |
| ESR (mm/h), n (%)  mean±SD  Median (min-max) | 18 (86)  33.5±22.2  34 (5-75) | 15 (71)  13.7±7.3  14 (2-31) | 14 (67)  * normal (14/14)  * (< 15) | 17 (81)  * normal (17/17)  * (< 15) |
| Physician PGA (0-10), n (%)  mean±SD  Median (min-max) | 21 (100)  6.4±1.6  7 (2-9) | 21 (100)  3.4±2.0  3.5 (0-8) | 18 (86)  1.3±1.6  1 (0-5) | 21 (100)  0.7±0.8  0.2 (0-3) |
| Patient/Parent PGA (0-10), n (%)  mean±SD  Median (min-max) | 21 (100)  6.3±2.0  7 (3-10) | 21 (100)  3.1±2.0  3 (0-8) | 18 (86)  1.8±1.7  2 (0-5) | 21 (100)  0.8±1.0  0.2 (0-3) |
| JADAS-10, n (%)  mean±SD  Median (min-max) | 21 (100)  21.4±6.0  22.5 (10-31) | 21 (100)  9.1±6.4  8.1 (1-24) | 18 (86)  3.5±3.5  3 (0-11) | 21 (100)  1.5±1.7  1.5 (0-7) |
| * CRP and ESR often tested externally and reported in the internal documentation as ‚normal‘ rather than numerical, therefore mean and median not calculable | | | | |

**Supplementary Table S2**

Joints with highest ultrasound activity building the MUSS at baseline (Oligoarthritis)

|  |  | **Highest B-Mode Activity** | | **Highest Doppler-Mode Activity** | |
| --- | --- | --- | --- | --- | --- |
| **Patient Number** |  | **Joints** | **Scores** | **Joints** | **Scores** |
| 1 |  | Knee | 3 | Knee | 3 |
| 2 |  | Tibiotalar | 2 | - | 0 |
| 3 |  | Tibiotalar | 1 | - | 0 |
| 4  5  6  7  8  9  10  11  12 |  | Tibiotalar  Knee  Knee  Elbow  Knee  Subtalar  Knee  Tibiotalar  Knee | 2  3  3  3  3  3  2  3  2 | Subtalar  Knee  Knee  Elbow  Knee  Subtalar  Knee  Tibiotalar  Knee | 3  3  2  2  2  3  2  2  2 |
| Notes: B-Mode and Doppler-Scores were attributed according to the pediatric OMERACT score, which grades findings from 0-3 | | | | | |

**Supplementary Table S3**

Joints with highest ultrasound activity building the MUSS at baseline (Polyarthritis)

|  |  | **Highest B-Mode Activity** | | **Highest Doppler-Mode Activity** | |
| --- | --- | --- | --- | --- | --- |
| **Patient Number** |  | **Joints** | **Scores** | **Joints** | **Scores** |
| 1 |  | Knee | 2 | Talonavicular | 1 |
| 2 |  | Knee | 2 | - | 0 |
| 3 |  | Knee | 3 | Knee | 3 |
| 4  5  6  7  8  9  10  11  12  13  14  15  16  17  18  19  20  21 |  | Subtalar  Knee  Hip/Shoulder  Hip/Tibiotalar/ Knee  Tibiotalar  Knee/Elbow  Knee  Wrist  Knee  Elbow/Tibiotalar  Subtalar  Hip  Hip/Knee/Elbow  Knee  Wrist  Knee  Tibiotalar/Subtalar  Tibiotalar/Talo-navicular | 2  2  2  3  3  3  3  2  3  1  2  3  3  3  2  2  2  3 | Subtalar  -  Shoulder  Tibiotalar  -  Knee  Knee  Wrist  Knee  Elbow/Tibiotalar  Subtalar  Subtalar  Wrist  Knee  Wrist  Knee  Subtalar  Talo-navicular | 2  0  3  2  0  2  2  3  2  1  2  2  3  1  2  3  2  2 |
| Notes: B-Mode and Doppler-Scores were attributed according to the pediatric OMERACT score, which grades findings from 0-3 | | | | | |

**Supplementary Table S4**

|  | **Oligoarthritis** | | | | | **Polyarthritis** | | | | |
| --- | --- | --- | --- | --- | --- | --- | --- | --- | --- | --- |
|  | T0 | T1 | T2 | T3 | Total n (%) | T0 | T1 | T2 | T3 | Total n (%) |
| **Patient, n (%)** | 12 (100) | 8 (67) | 7 (58) | 10 (83) | 37 (100) | 21 (100) | 21 (100) | 18 (86) | 21 (100) | 81 (100) |
| **Knee joint** | | | | | | | | | | |
| Patient n (%) | 12 (100) | 8 (100) | 7 (10) | 10 (100) | 37 (100) | 21 (100) | 21 (100) | 17 (94) | 20 (95) | 79 (98) |
| Total joints scanned n | 24 | 16 | 14 | 20 | 74 (100) | 42 | 42 | 34 | 40 | 158 |
| **Ankle joint** | | | | | | | | | | |
| Patient n (%) | 12 (100) | 7 (88) | 7 (100) | 10 (100) | 36 (97) | 21 (100) | 21 (100) | 18 (100) | 21 (100) | 81 (100) |
| Total joints scanned n | 24 | 14 | 14 | 20 | 72 (97) | 42 | 42 | 36 | 42 | 162 |
| **Midfoot** (Talo-navicular and calcaneo-navicular joints) | | | | | | | | | | |
| Patient n (%) | 12 (100) | 7 (88) | 7 (100) | 10 (100) | 36 (97) | 21 (100) | 20 (100) | 18 (100) | 19 (90) | 78 (96) |
| Total joints scanned n | 24 | 14 | 14 | 20 | 72 (97) | 42 | 40 | 36 | 38 | 156 |
| **Hip joint** | | | | | | | | | | |
| Patient n (%) | 8 (67) | 4 (50) | 5 (71) | 4 (40) | 21 (57) | 21 (100) | 18 (86) | 13 (72) | 18 (86) | 70 (86) |
| Total joints scanned n | 16 | 8 | 10 | 8 | 42 (57) | 42 | 36 | 26 | 36 | 140 |
| **Elbow joint** | | | | | | | | | | |
| Patient n (%) | 6 (50) | 5 (63) | 1 (14) | 2 (20) | 14 (38) | 17 (81) | 11 (52) | 7 (39) | 7 (33) | 42 (52) |
| Total joints scanned n | 12 | 10 | 2 | 4 | 28 (38) | 34 | 22 | 14 | 14 | 84 |
| **Wrist joint** (radio-carpal, medio-carpal and radio-ulnar joints) | | | | | | | | | | |
| Patient n (%) | 5 (42) | 4 (50) | 1 (14) | 1 (10) | 11 (30) | 17 (81) | 11 (52) | 9 (50) | 8 (38) | 45 (56) |
| Total joints scanned n | 10 | 8 | 2 | 2 | 22 (30) | 34 | 22 | 18 | 16 | 90 |
| **Shoulder joint** | | | | | | | | | | |
| Patient n (%) | 1 (8) | 1 (13) | 0 | 0 | 2 (5) | 6 (29) | 4 (19) | 2 (11) | 2 (10) | 14 (17) |
| Total joints scanned n | 2 | 2 | 0 | 0 | 4 (5) | 12 | 8 | 4 | 4 | 28 |
| **Total joints scanned n**  **%** | 112/168  67 | 72/112  64 | 56/98  57 | 74/140  53 | 314  61 | 248/294  84 | 212/294  72 | 168/252  67 | 190/294  65 | 818  72 |
| ^1^**BM-score,**  mean +SD  median (min-max) | 2.5±0.6  3 (1-3) | 0.2±0.4  0 (0-1) | 0 (0) | 0 (0) |  | 2.4±0.6  2 (1-3) | 0.9±1.1  0 (0-3) | 0.3±0.7  0 (0-3) | 0.1±0.5  0 (0-2) |  |
| ^1^**PD-score**  mean +SD  median (min-max) | 1.9±1.0  2 (0-3) | 0 (0) | 0 (0) | 0 (0) |  | 1.8±1.0  2 (0-3) | 0.8±1.0  0 (0-3) | 0.3±0.7  0 (0-3) | 0±0.2  0 (0-1) |  |
| **MUSS**  mean +SD  median (min-max) | 4.4±1.5  5 (1-6) | 0.2±0.4  0 (0-1) | 0 (0) | 0 (0) |  | 4.2±1.2  5 (2-6) | 1.8±2.1  1 (0-6) | 0.5±1.4  0 (0-6) | 0.2±0.6  0 (0-3) |  |
| *All joints were scanned symmetrically for each patient at each point. BM: B-Mode, PD: power Doppler, MUSS: Musculoskeletal Ultrasound Sum Score. ^1^The highest BM and PD score per patient from all their individual joints scanned was used for the analysis. Scores were attributed according to the pediatric OMERACT score, which grades findings from 0-3. Due to the low level of affection, finger and toe joints were not analyzed. | | | | | | | | | | |
|  |  |  |  |  |  |  |  |  |  |  |

Summary of the individual joints scanned per patient per follow-up using musculoskeletal ultrasound
